# Comparative performance of Filamentous Fungi 4.0 and Mass Spectrometry Identification 2.0 databases for *Aspergillus* spp.: identification using a simplified protein extraction method

**DOI:** 10.1590/S1678-9946202668014

**Published:** 2026-02-16

**Authors:** Gustavo Giacon Damiani, Vivian Caso Coelho, Juliana Possato Fernandes Takahashi, Ingrid Gonçalves Costa Leite, Marcia Regina von Zeska Kress, Marcello Mihailenko Chaves Magri, Valdes Roberto Bollela, Roberto Martínez, Gil Benard, Tiago Alexandre Cocio

**Affiliations:** 1 Universidade de São Paulo, Faculdade de Medicina, Instituto de Medicina Tropical de São Paulo, Laboratório de Investigação Médica em Micologia (LIM-53), São Paulo, São Paulo, Brazil; 2 Universidade de São Paulo, Faculdade de Medicina, Hospital das Clínicas, Laboratório de Investigação Médica em Imunologia (LIM-48), São Paulo, São Paulo, Brazil; 3 Instituto Adolfo Lutz, Divisão de Patologia, São Paulo, São Paulo, Brazil; 4 Universidade Estadual Júlio de Mesquita Filho, Programa de Pós-Graduação em Doenças Tropicais, Botucatu, São Paulo, Brazil; 5 Universidade de São Paulo, Faculdade de Ciências Farmacêuticas de Ribeirão Preto, Departamento de Análises Clínicas, Toxicológicas e Bromatológicas, Ribeirão Preto, São Paulo, Brazil; 6 Universidade de São Paulo, Faculdade de Medicina, Hospital das Clínicas, Departamento de Doenças Infecciosas e Parasitárias, São Paulo, São Paulo, Brazil; 7 Universidade de São Paulo, Faculdade de Medicina de Ribeirão Preto, Departamento de Clínica Médica, Ribeirão Preto, São Paulo, Brazil

**Keywords:** *Aspergillus* identification, MALDI-TOF MS, Spectral databases, Medical mycology

## Abstract

*Aspergillus* spp. are major agents of pulmonary aspergillosis, posing diagnostic challenges in clinical laboratories due to morphological similarity among species. This study compared the performance of two spectral databases, Filamentous Fungi v4.0 (Bruker Daltonics) and Mass Spectrometry Identification v2.0 (MSI 2.0), for *Aspergillus* species identification by MALDI-TOF MS, and evaluated the applicability of the simplified protein extraction method from solid culture medium for routine use in hospital laboratories. Overall, 46 clinical isolates from patients with pulmonary aspergillosis were cultured on Sabouraud Dextrose Agar (SDA) at 37 °C for 48 h. Proteins were extracted directly from colonies and analysed using the Bruker MALDI Biotyper system. Spectra were compared against FF_4.0 and MSI_2.0 databases, and results were correlated with molecular identification by *ben*A, *cmd*5, and ITS gene sequencing. The extraction method yielded high-quality spectra with peaks between 2–15 kDa. The FF_4.0 database identified 52.17% of isolates at the section level and none at the species level, reflecting limited spectral compatibility and taxonomic coverage. Conversely, MSI_2.0 correctly identified 82.6% of isolates at the species level, 15.23% at the section level, and only 2.17% were not identified. Concordance between MSI_2.0 and sequencing reached 44.7%, without statistical significance (p = 0.627). The simplified solid-medium extraction method combined with MSI 2.0 proved efficient, reproducible, and suitable for clinical routine, offering faster identification of *Aspergillus* species compared to conventional methods. In contrast, FF_4.0 showed limited applicability for hospital workflows.

## INTRODUCTION

Pulmonary aspergillosis, caused by *Aspergillus* spp., is a systemic mycosis mainly affecting individuals with immune dysfunction, preexisting respiratory diseases and rarely in immunocompetent patients. The clinical forms of the diseased include chronic pulmonary aspergillosis (CPA), often linked to cystic fibrosis (CF), post-pulmonary tuberculosis (PTB), or nontuberculous mycobacterial pulmonary disease (NTM-PD)^1–4^ and acute invasive aspergillosis (AIA)^5^, common in immunosuppressed patients such as transplant recipients and cancer patients. More recently, coinfection with severe acute respiratory syndrome coronavirus 2 (SARS-CoV-2), known as COVID-19-associated pulmonary aspergillosis (CAPA) has emerged, showing additional challenges in diagnosis and treatment^6^.

The identification and isolation of *Aspergillus* spp. from respiratory samples of these patients, remains a major challenge for medical microbiology laboratories. Conventional techniques species identification, such as morphological analysis, biochemical assays, serological tests, and sequencing, have considerable limitations, as many species share similar characteristics, making their precise differentiation difficult^7,8^. Furthermore, the slow fungal growth of some genera, sample preparation, and delays in this laboratories results contribute to late and sometimes inaccurate diagnoses, which may compromise appropriate therapeutic decisions^9^.

Matrix-assisted laser desorption/ionization time-of-flight mass spectrometry (MALDI-TOF MS) has emerged as a valuable tool for the identification of microorganisms offering a rapid, cost-effective, and accurate protein-based analysis that supports clinical diagnosis^9,10^. Considering the clinical importance of *Aspergillus* spp. identification at the species level, particularly in hospital environments, it is essential to evaluate the reliability of current MALDI-TOF MS methods^11^. Previous studies have shown that database performance may vary depending on the protein extraction method and spectral library used^12^. Nonetheless, existing databases still face limitations since incomplete or overlapping spectral profiles among closely related species can result in misidentification or low confidence scores^11,12^. These challenges are especially relevant for *Aspergillus* spp. complexes, in which accurate identification requires comprehensive reference spectra and standardized extraction protocols^13^.

Although, gene sequencing (e.g., of Internal Transcribed Spacer (ITS), β-tubulin (*ben*A), calmodulin (*cmd*5)) remains the gold standard for definitive *Aspergillus* species identification^14–16^, thus MALDI-TOF MS permits rapid protein-spectral profiling from cultured isolates, enabling species-level identification in many cases much faster than sequencing^9–13,17,^. In practice, MALDI-TOF MS and sequencing complement each other: the first method can be an efficient screening or medical routine, while gene sequencing continues to play a critical confirmatory role^9–13,17^.

This work evaluated protein extraction on solid medium (Sabouraud Dextrose Agar, SDA) as described by Li *et al.* ^18^ from application in routine medical laboratory and also compare the accuracy of the Filamentous Fungi v4.0 database (FF_4.0) (Bruker Daltonics, Bremen, Germany) and the Mass Spectrometry Identification v2.0 (MSI_2.0) platform using spectra acquired on the Bruker spectrophotometer platform correlating the results with *ben*A gene sequencing, using *Aspergillus* spp. from patients in tertiary hospital in a city in Sao Paulo State, Brazil.

## MATERIALS AND METHODS

### Ethics

The study protocol was reviewed and approved by the Research Ethics Committee of the Hospital das Clinicas de Ribeirao Preto, Ribeirao Preto Medical School, University of Sao Paulo (HC-FMRP/USP), under process N° CAAE: 54893722.1.0000.5440.

### *Aspergillus* spp. strains

Forty-six *Aspergillus* spp. isolates collected from 2001 to 2021 were obtained from patients treated at the HC-MRP/USP, with pulmonary aspergillosis associated with PTB, NTM, or CF, as well as AIA (including COVID-19–associated cases) (Table 1).

**Table 1:** Identification results and score values of *Aspergillus* spp. clinical isolates obtained from molecular sequencing (*ben*A, *cmd*5, and ITS genes) and spectral databases (FF_4.0 and MSI_2.0) with biological concordance between molecular biological method and spectral database MSI_2.0.

			Filamentous Fungi 4.0 (FF_4.0)		Mass Spectrometry Identification (MSI _2.0)		Biological species concordance between sequencing and MSI_2.0
Strains ID	Molecular identification	GenBank number	Scores	Section	Index	Species/Section	False or true only in Index A identification
***LMC6002.01***	*A. neoniger*	OR100836.1	1.45	*A. niger* *	**A**	*A. tubingensis*	False
***LMC6028.01***	*A. parasiticus*	OR100836.1	1.27	*A. parasiticus* *	**A**	*A. oryzae*	False
***LMC6009.01***	*A. parasiticus*	OR100831.1	1.16	*A. flavus group* *	**A**	*A. oryzae*	False
***LMC6010.02***	*A. parasiticus*	OR100832.1	1.30	*A. flavus group* *	**A**	*A. oryzae*	False
***LMC6010.01***	*A. fumigatus*	OR100789.1	1.83	*A. fumigatus*	**B**	*A. fumigatus*	-
***LMC6011.01***	*A. fumigatus*	OR100790.1	1.84	*A. fumigatus*	**B**	*A. fumigatus*	-
***LMC6012.01***	*A. parasiticus*	OR100833.1	1.24	*A. parasiticus* *	**A**	*A. novoparasiticus*	False
***LMC6017.01***	*A. terreus*	-	*-*	**Unidentified Species** *	**A**	*A. alabamensis*	False
***LMC6018.01***	*A. fumigatus*	OR100797.1	1.87	*A. fumigatus*	**B**	*A. fumigatus*	-
***LMC6022.01***	*A. flavus*	OR100784.1	1.70	*A. flavus group*	**A**	*A. flavus/oryzae*	False
***LMC6025.01***	*A. fumigatus*	OR100801.1	1.70	*A. fumigatus*	**A**	*Aspergillus sect.* Fumigati	False
***LMC6030.01***	*A. flavus*	OR100787.1	1.70	*A. flavus group*	**A**	*A. flavus/oryzae*	False
***LMC6017.02***	*A. fumigatus*	OR100795.1	1.72	*A. fumigatus*	**A**	*A. fumigatus*	True
***LMC6017.03***	*A. fumigatus*	OR100796.1	1.76	*A. fumigatus*	**B**	*A. fumigatus*	-
***LMC9001.01***	*A. flavus*	OR225627.1	1.72	*A. flavus group*	**A**	*A. flavus/oryzae*	False
***LMC9002.01***	*A. flavus*	OR225628.1	1.70	*A. flavus group*	**A**	*A. flavus/oryzae*	False
***LMC9003.01***	*A. fumigatus*	OR100811.1	1.79	*A. fumigatus*	**B**	*A. fumigatus*	-
***LMC9004.01***	*A. fumigatus*	OR100812.1	1.74	*A. fumigatus*	**A**	*A. fumigatus*	True
***LMC9005.01***	*A. flavus*	OR225629.1	1.60	*A. flavus group* *	A	*A. flavus/oryzae*	False
***LMC9007.01***	*A. flavus*	OR225631.1	1.64	*A. flavus group* *	**A**	*A. flavus/oryzae*	False
***LMC9009.01***	*A. fumigatus*	OR100814.1	1.88	*A. fumigatus*	**B**	*A. fumigatus*	-
***LMC9010.01***	*A. flavus*	OR225632.1	1.32	*A. flavus group* *	**C**	*A. sojae*	-
***LMC9011.01***	*A. flavus*	OR225633.1	1.72	*A. flavus group*	**A**	*A. flavus/oryzae*	False
***LMC9011.02***	*A. flavus*	-	1.72	*A. flavus group*	**A**	*A. flavus/oryzae*	False
***LMC9013.01***	*A. fumigatus*	OR100815.1	1.85	*A. fumigatus*	**A**	*A. fumigatus*	True
***LMC9018.01***	*A. fumigatus*	OR100820.1	1.62	*A. fumigatus* *	**A**	*A. fumigatus*	True
***LMC9022.01***	*A. fumigatus*	OR100824.1	1.66	*A. fumigatus* *	**A**	*A. fumigatus*	True
***LMC9024.01***	*A. fumigatus*	OR100826.1	1.51	*A. fumigatus* *	**A**	*A. fumigatus*	True
***LMC9025.01***	*A. fumigatus*	OR100827.1	1.75	*A. fumigatus*	**A**	*A. fumigatus*	True
***HCRP260***	*A. parasciticus*	PQ365496	1.30	*A.parasiticus* *	**A**	*A. oryzae*	False
***HCRP261***	*A. tamarii*	PV822540	1.20	*A. tamarii* *	**A**	*A. tamarii*	True
***HCRP262***	*A. tamarii*	PV822541	1.15	*A. flavus/oryzae* group*	**A**	*A. tamarii*	True
***HCRP264***	*A. fumigatus*	PV996901	1.90	*A. fumigatus*	**A**	*A. fumigatus*	True
***HCRP267***	*A. fumigatus*	PV996902	1.90	*A. fumigatus*	**A**	*A. fumigatus*	True
***HCRP271***	*A. fumigatus*	PV996903	1.75	*A. fumigatus*	**A**	*A. fumigatus*	True
***HCRP294***	*A. flavus*	PV996908	1.53	*A. flavus* *	**A**	*A. flavus/oryzae*	False
***HCRP298***	*A. flavus*	PV996909	1.88	*A. flavus*	**A**	*A. flavus/oryzae*	False
***HCRP303***	*A. terreus*	PV996913	1.65	*A. terreus* *	**A**	*A.terreus*	True
***HCRP307***	*A. flavus*	PV996910	1.84	*A. flavus*	A	*A. flavus/oryzae*	False
***HCRP308***	*A. flavus*	PV996911	1.82	*A. fumigatus*	**A**	*A. flavus/oryzae*	False
***HCRP309***	*A. flavus*	PV996912	1.60	*A. flavus* *	**A**	*A. flavus/oryzae*	False
***HCRP314***	*A. fumigatus*	PV996904	1.39	*A. fumigatus* *	**A**	*A. fumigatus*	True
***HCRP323***	*A. welwitschiae*	PQ558662	1.06	*A. niger* *	**B**	*A. niger*	-
***HCRP325***	*A. fumigatus*	PV996905	1.65	*A. fumigatus* *	**A**	*A. fumigatus*	True
***HCRP330***	*A. fumigatus*	PV996906	1.50	*A. fumigatus* *	**A**	*A. fumigatus*	True
***HCRP340***	*A. fumigatus*	PV996907	1.70	*A. fumigatus*	**A**	*A. fumigatus*	True

LMC isolates = Laboratory of Clinical Mycology and Fungal Gene Expression and Proteomics, Department of Clinical, Toxicological and Bromatological Analyses, School of Pharmaceutical Sciences of Ribeirao Preto, University of Sao Paulo (LMC-FCFRP/USP); HCRP isolates = Hospital das Clinicas de Ribeirao Preto, Ribeirao Preto Medical School, University of Sao Paulo (HC-FMRP/USP); criteria of spectra's database =: MSI_2.0 identification indices; A = species-level identification; B = genus-level identification; C = not identified. FF_4.0 identification score criteria: 3.0–2.0 for species-level identification; 1.99–1.70 for genus-level identification; and ≤ 1.69 for not identified;

* *Aspergillus* spp. that obtained a score below 1.70 is considered, according to Bruker guidelines, as a non-identified species. In the report, the FF_4.0 database suggests the species that fall under this classification. Biological species concordance between sequencing and MSI_2.0, only in an Index A identification, is false when no species-level identification match is observed, and as true when both methods provide concordant species-level identification within the *Aspergillus* genus.

The identification of *Aspergillus* spp. strains was determined by sequencing *ben*A (β-tubulin), *cmd*5 (calmodulin), and ITS (regions 1 and 4) genes for each isolate evaluated, which were obtained from previous identifications: LMC's strains were sequenced by Fonseca *et al.* ^19^, and HCRP strains were sequenced in prior studies (Cocio *et al*., unpublished data) (Table 1).

### Protein extraction of *Aspergillus* spp. strain

MALDI-TOF MS analysis was performed following protein extraction on solid medium (SDA), according to Li *e al.* ^18^. Clinical isolates and reference strains (ATCC 204304: *A. flavus*, e ATCC 46645: *A. fumigatus*) were grown on 60×15 mm plates (Kasvi) for 48 h at 37 °C, after this step, the conidia were collected. For protein extraction from the conidia of each *Aspergillus* spp. included in this study, 25 μL of 70% formic acid was added followed by 25 μL of 100% acetonitrile, with a totalization of 10 min incubation. After centrifugation, 1 μL of the supernatant was applied onto the MSP 96 Polished Steel Target Plate (Bruker Daltonik GmbH, Bremen, Germany), air-dried and overlaid with 1 μL of HCCA matrix (α-cyano-4-hydroxycinnamic acid) reconstituted in 50% acetonitrile and 1.5% TFA (trifluoroacetic acid), then allowed to dry again.

### Acquisition of protein spectra of *Aspergillus* spp.

Spectra from each *Aspergillus* spp. evaluated in this study, were acquired on a Bruker IVD MALDI Biotyper Microflex LT/SH^®^ (Bruker Daltonik GmbH, Bremen, Germany) at the Clinical Microbiology Laboratory of HCFMRP/USP, with the laser operated under the manufacturer's factory default settings.

All *Aspergillus* spp. spectra obtained were analysed in the software FlexControl 3.4 and MALDI Biotyper RTC (Bruker Daltonics, Bremen, Germany) to verify the quality of the spectra by examining (m/z) peak relationships.

### Comparative accuracy assessment of the evaluated spectral databases for the identification of *Aspergillus* spp. by MALDI-TOF MS

For the identification and comparative analysis of spectral data base, all *Aspergillus* spp. spectra obtain was used in the FF_4.0 (Bruker Daltonics, Bremen, Germany) and the MSI_2.0 online platform developed by Normand *et al.* ^10^ observed and evaluated the values of score and indices showed.

For analysis the FF_4.0 data base, we applied the score cutoffs described by Bruker Daltonics, which were as follows: 3.0 to 2.0 for species level identification; 1.99 to 1.70 for section level identification; and 1.69 or score below were not identified.

For MSI_2.0 analysis, we followed the score classification indices indicated by platform^10^ (A – species-level identification; B – section-level identification; C – not identified).

A descriptive statistical analysis based on frequency and proportion was performed to compare the efficiency of the spectral databases (FF_4.0 and MSI_2.0) in the identification of *Aspergillus* spp. clinical isolates. The results were expressed as absolute (n) and relative (%) frequencies according to the level of identification achieved by each database (species level, genus level, or no identification). This approach enabled the assessment of the databases’ performance in accurately identifying isolates at different taxonomic levels.

### Correlation between the effectiveness of the evaluated spectral databases and sequencing of *Aspergillus* spp.

The analyses generated by both spectral databases, evaluated in this study, correlated this data with the sequencing data of the *ben*A, *cmd*5, and ITS gene from each *Aspergillus* spp. strain (Table 1) as described elsewhere.

To assess the efficiency of the databases in identifying species of the *Aspergillus* genus, a two-tailed statistical test was performed to verify the biological concordance between genetic sequencing and the spectral database (MALDI-TOF MS). As a criterion, any spectral database that was not effective in species-level identification was excluded from the analysis.

## RESULTS

**Figure 1:**
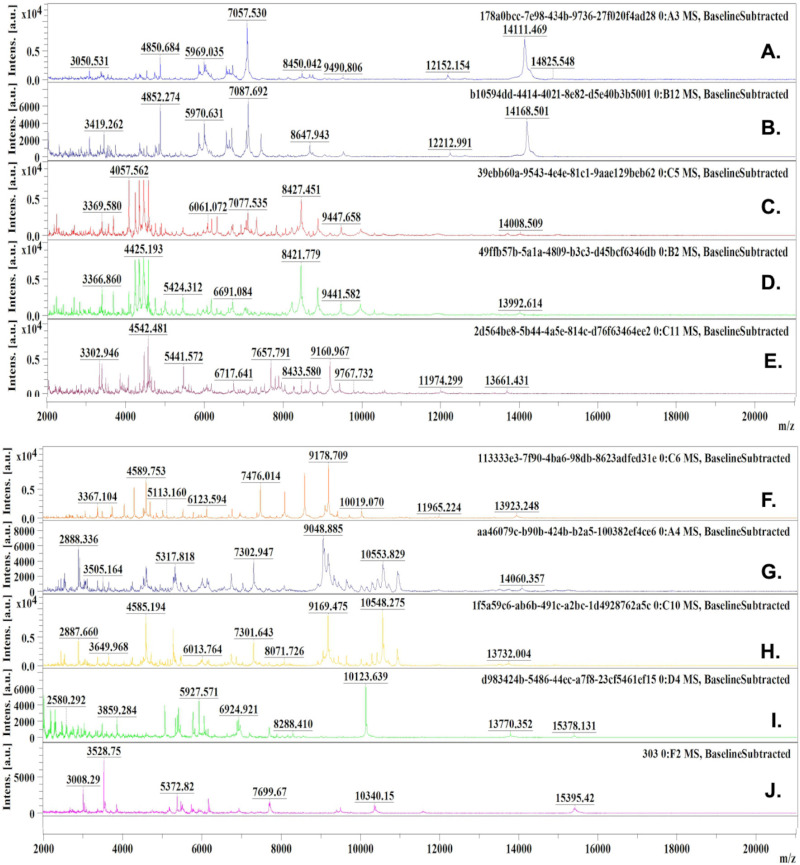
Protein spectra generated from *Aspergillus* spp. strains in Sabouraud dextrose agar (SDA) grown, using the protein extraction method described by Li *et al.* ^18^: (A) HCRP141 (LMC.6017.02)*A. fumigatus*; (B) ATCC 46645 – *A. fumigatus*; (C) ATCC 204304 – *A. flavus*; (D) HCRP307 – *A. flavus*; € HCRP260 – *A. parasiticus*; (F) HCRP124 (LMC6017.01) – *A. terreus*; (G) HCRP303 – *A. terreus*; (H) HCRP124 – *A. terreus*; (I) HCRP65 – *A. neoniger*; (J) HCRP323 – *A. niger*.

The protein extraction method based for *Aspergillus* spp. on growth on solid culture medium, proved effective for acquiring protein spectra from clinical isolates belonging to the Fumigati, Flavi, Terrei, and Nigri sections, respectively. Fungal growth on Sabouraud Dextrose Agar (SDA) medium at 37 °C for 48 h, yielded well defined protein spectra with clearly resolved peaks and mass-to-charge (m/z) values. Putative proteins with m/z values ranging from 2,000 to 15,000 *kilodaltons* (≈2–15 kDa) were observed in the clinical and ATCC isolates analysed in this study (Figure 1).

Analysis of the protein spectra, with the FF_4.0 database did not identify any of the 46 *Aspergillus* spp. isolates at the species level (Tables 1 and 2). At the section level, however, it classified 24 *Aspergillus* isolates, 52.2% of all samples with score values ranging from 1.90 to 1.70 (Table 2). A total of 22 clinical isolates were reported as "no identification," totalling 47.8%, with score ranging from1.65 to 1.06 (Table 2). These results indicate that the FF_4.0 spectral database was not efficient for species-level identification of *Aspergillus* spp. using the protein extraction method from SAB solid culture. Nevertheless, the MSI_2.0 spectral database could identify 38 82.6% of the *Aspergillus* spp. Ate the species level (Table 2). Seven (15.23%) isolates were classified only at the genus level. Only a single isolate could not be identified (Table 2). Thus, MSI_2.0 proved effective for identifying *Aspergillus* spp..

**Table 2:** Comparative identification performance of the FF_4.0 and MSI_2.0 spectral databases and Statistical analysis of biological concordance between molecular sequencing and MSI_2.0 spectral identification of *Aspergillus* spp. clinical isolates.

Spectral database / Statistical test	Identification level / Parameter	Number of isolates (n) / Observed concordance	Percentage (%) / Expected concordance	Score range / p-value	Interpretation
**FF_4.0**	Species level	0	0.00	–	Inefficient at species-level identification
	Genus’ level	24	52.17	1.90 – 1.70	Moderate efficiency at genus level
	No identification	22	47.83	1.65 – 1.06	Poor database performance
**MSI_2.0**	Species level (Index A)	38	82.60	–	High identification efficiency
	Genus’ level (Index B)	7	15.23	–	Moderate genus-level classification
	No identification (Index C)	1	2.17	–	Lower effective; minimal non-identification
**Two-tailed exact binomial test (MSI_2.0 vs. molecular sequencing)**	Biological species concordance	17 concordant (44.7%) / 21 discordant (55.3%)	50% concordance expected	0.627	No statistically significant difference: observed variation may be due to chance

FF_4.0 = spectral database with score criteria of 3.0–2.0 for species-level identification, 1.99–1.70 for genus-level identification, and ≤ 1.69 for unidentified isolates; MSI_2.0: MALDI-TOF spectral database using identification indexes A (species level), B (genus level), and C (unidentified). A total of 46 *Aspergillus* spp. clinical isolates were analyzed using the protein extraction method from Sabouraud (SAB) solid culture. The MSI_2.0 database showed markedly higher identification efficiency compared with FF_4.0, particularly at the species level (82.6% vs. 0%). The two-tailed exact binomial test was used to evaluate whether the proportion of biological concordance between molecular sequencing (*ben*A, *cmd*5, ITS) and the MSI_2.0 spectral database differed significantly from a 50% expected distribution. The resulting p-value (0.627) indicated that the observed proportions of concordant and discordant identifications were not statistically different (p > 0.05), suggesting random variation rather than systematic bias between the two identification methods.

Upon comparing the molecular sequencing results (*ben*A, *cmd*5, and ITS genes) with the MSI_2.0 spectral database identification, biological concordance between the two methods was observed in 17 out of 38 clinical isolates (44.7%), while 21 isolates (55.3%) showed discordance at the species level (Table 2). A two-tailed exact binomial test was applied to evaluate whether the observed concordance differed significantly from an expected 50% distribution. The analysis yielded a *p-value* of 0.627, indicating no statistically significant difference between the proportion of concordant and discordant results (Table 2). Although molecular sequencing provided species-level resolution for all isolates, the MSI_2.0 database achieved genus-level or partial identifications in some cases, especially among closely related taxa such as *A. flavus* and *A. oryzae* (Table 2). These results suggest that, while MSI_2.0 demonstrates satisfactory performance for most *Aspergillus* isolates, its ability to discriminate species within the same complex remains limited.

## DISCUSSION

This study compared the performance of the FF_4.0 (Bruker Daltonics) and MSI_2.0 databases for identifying clinical isolates of *Aspergillus* spp. by MALDI-TOF MS and evaluated the applicability of the protein extraction method described by Li *et al.* ^18^ for routine use in hospital laboratories.

The method proposed by Li *et al.* ^18^ stood out for its simplicity, reproducibility, and rapid execution, fundamental features in hospital environments where diagnostic agility is critical for managing fungal infections. The protocol, based on colonies cultivated on SDA at 37 °C for 48 h, produced well-defined spectra between 2 and 15 kDa indicating the high quality achievable with the protein extraction method (Figure 1). These findings are consistent with Shao *et al.* ^9^, who emphasized that standardized extraction procedures are crucial to ensure spectral quality and reproducibility. Extraction protein from young colonies proved suitable for clinical laboratories, eliminating complex steps, and reducing operational costs without compromising spectral definition.

Despite the quality of the obtained spectra, the FF_4.0 database showed limited performance, identifying 52.17% of isolates at the section level and none at the species level (Table 2). This limitation primarily reflects differences between the extraction conditions used in this study and those applied during the database's construction. The FF_4.0 spectral library was developed using reference strains cultivated at 25–30 °C in liquid medium, favoring extraction of stable intracellular proteins and generating homogeneous spectra^18^. Conversely, the protocol applied in this study includes cell wall and secreted proteins expressed at physiological temperature, producing distinct proteomic profiles that reduce spectral similarity and, consequently, identification scores.

Another factor contributing to the poor performance of the Bruker library is its limited taxonomic coverage. Although version 4.0 includes 222 species or fungal groups, only 27 species represent the genus *Aspergillus* ^12^. Studies by Vidal-Acuña *et al.* ^17^ and Wilkendorf *et al.* ^13^ demonstrated that the limited number of reference strains and the predominance of ATCC isolates reduce spectral diversity and, therefore, the discriminatory capacity of the Filamentous Fungi library. Possibly, the Bruker Biotyper matching algorithm, based on dominant peaks (Main Spectra Profiles), is highly sensitive to minor variations in mass or intensity, penalizing spectra obtained under differing experimental conditions^19^. This methodological rigidity limits adaptability to simplified protocols, making the library less suitable for hospital routine use.

In contrast, the MSI_2.0 database showed notably better performance, identifying 82.6 % of isolates at the species level, 15.23 % at the section level, and only 2.17 % as unidentified (Table 2), which aligns with the multicenter study by Normand *et al*.^10^, in which MSI-2 achieved 83.25% correct identifications among 633 filamentous fungal isolates, markedly outperforming both Bruker and its previous version. The MSI 2.0 spectral database comprises 1,031 reference spectra representing 358 fungal species, including 154 spectra belonging to the genus *Aspergillus* ^10^. The success of MSI 2.0 identification is attributed to its collaborative curation and continuous updates, integrating spectra obtained under diverse growth conditions, media, and temperatures and applying more flexible matching algorithms. Another important aspect is that MSI 2.0 provides broad coverage of *Aspergillus* species, including cryptic complexes and resistant isolates from Fumigati, Flavi, Nigri, and Terrei sections. Thus, combining the simplified extraction method with the MSI 2.0 library offers a practical and efficient approach for identifying *Aspergillus* spp. directly from cultured colonies, significantly reducing diagnostic turnaround time.

Comparison between MSI 2.0 and molecular sequencing of the *ben*A, *cmd*5, and ITS genes revealed 44.7% biological concordance and 55.3 % biological discordance, without statistical significance (p = 0.627) (Table 2). Although sequencing remains the gold standard, MALDI-TOF MS offers clear operational advantages: speed, lower cost, and suitability for clinical workflows. According to Schoch *et al.* ^14^ and Samson *et al.* ^15^, accurate molecular identification of *Aspergillus* requires multiple genetic markers to resolve cryptic species, which increases analysis time and cost. Therefore, implementing MALDI-TOF MS as an initial screening tool followed by molecular confirmation of inconclusive results represents an efficient and balanced model for hospital laboratories.

The limitation of this study was the relatively small number of clinical isolates analyzed (n = 46), all obtained from a single hospital center, which restricts the representativeness and generalization of the results to other populations and laboratory settings. Furthermore, the discrepancy observed between the identifications obtained by MALDI-TOF MS, particularly with the MSI_2.0 database, and the gene sequencing results, with only 44.7% concordance between the methods, highlights the limitation of the spectrometric method in differentiating cryptic species belonging to phylogenetically related complexes, such as *A. flavus/A. oryzae* and species from the Nigri section. Note that only one protein extraction protocol (based on growth on solid medium) and two spectral databases were evaluated, which may have influenced the comparative performance and limit the generalization of the findings regarding the method's applicability under different laboratory conditions. Previous studies have shown that the performance of MALDI-TOF MS databases can vary according to several analytical conditions, particularly the protein extraction method, the culture conditions used for biomass production, the quality and quantity of biological material, and the parameters applied during spectral acquisition. In this context, González-Lara *et al*.^12^ reported superior performance of the MSI_2.0 database under certain conditions, reinforcing that differences in extraction protocols and in the representation or update level of spectral libraries can significantly affect the identification results. Therefore, the interpretation of the findings should consider that methodological variations, especially those related to protein extraction and database composition, may explain discrepancies observed between studies. These factors highlight the need for methodological standardization and continuous evaluation of available spectral databases, particularly for genera with high spectral diversity such as *Aspergillus* spp.

## CONCLUSION

In summary, the protein extraction method proposed by Li *et al.* ^18^, combined with the MSI_2.0 database, proved to be an efficient, economical, and feasible strategy for identifying *Aspergillus* spp. in hospital routine laboratories. Also, in a future study, there is a possibility of constructing a spectral database using the protein extraction methodology evaluated in this work, which may assist in the identification of cryptic species within the genus *Aspergillus*.

## Data Availability

The complete anonymized dataset supporting the findings of this study is included within the article itself.
